# The swine flu vaccine, public attitudes, and researcher interpretations: a systematic review of qualitative research

**DOI:** 10.1186/s12913-016-1466-7

**Published:** 2016-06-24

**Authors:** Benedicte Carlsen, Claire Glenton

**Affiliations:** Uni Research Rokkan Centre, Nygaardsgt 112, N-5008 Bergen, Norway; Norwegian Institute of Public Health, PO Box 7004, St. Olavs plass, N-0130 Oslo, Norway

**Keywords:** Pandemic, Swine flu, Vaccination, H1N1, Qualitative research, Systematic review, Reflexivity, Research context

## Abstract

**Background:**

During pandemics, health authorities may be uncertain about the spread and severity of the disease and the effectiveness and safety of available interventions. This was the case during the swine flu (H1N1) pandemic of 2009–2010, and governments were forced to make decisions despite these uncertainties. While many countries chose to implement wide scale vaccination programmes, few accomplished their vaccination goals. Many research studies aiming to explore barriers and facilitators to vaccine uptake have been conducted in the aftermath of the pandemic, including several qualitative studies.

**Aims:**

1. To explore public attitudes to the swine flu vaccine in different countries through a review of qualitative primary studies.
2. To describe and discuss the implications drawn by the primary study authors.

**Methods:**

Systematic review of qualitative research studies, using a broadly comparative cross case-study approach. Study quality was appraised using an adaptation of the Critical Appraisal Skills Programme (CASP) quality assessment tool.

**Results:**

The review indicates that the public had varying opinions about disease risk and prevalence and had concerns about vaccine safety. Most primary study authors concluded that participants were uninformed, and that more information about the disease and the vaccine would have led to an increase in vaccine uptake. We find these conclusions problematic. We suggest instead that people’s questions and concerns were legitimate given the uncertainties of the situation at the time and the fact that the authorities did not have the necessary information to convince the public. Our quality assessment of the included studies points to a lack of reflexivity and a lack of information about study context. We suggest that these study weaknesses are tied to primary study authors’ lack of acknowledgement of the uncertainties surrounding the disease and the vaccine.

**Conclusion:**

While primary study authors suggest that authorities could increase vaccine uptake through increased information, we suggest instead that health authorities should be more transparent in their information and decision-making processes in future pandemic situations.

**Electronic supplementary material:**

The online version of this article (doi:10.1186/s12913-016-1466-7) contains supplementary material, which is available to authorized users.

## Background

Over the last few decades, global and national governments have issued a number of warnings to the general public about the outbreak of potentially dangerous diseases. There has been widespread concern that these diseases, including SARS and seasonal influenzas, could cause serious harm to individuals and could pose a threat to public health. However, there has also been uncertainty regarding the spread and severity of these diseases as well as the effectiveness and safety of available preventive or curative interventions. When faced with the possibility of widespread disease but with high degrees of uncertainty, governments need to make difficult decisions about how to respond. In some cases, they have chosen to deliver large-scale public health interventions, such as vaccines. However, the success of these strategies depends on public participation and support, and members of the public also need to make their own decisions while faced with the same uncertainties.

In this review, we synthesised existing studies of public reactions to the so-called swine flu pandemic of 2009–2010 and the vaccination strategies that were put in place. By synthesising the results of primary studies that have explored people’s attitudes to these strategies and the implications that primary study authors have drawn from these studies, we hoped to learn lessons that could be useful for future pandemics.

### The swine flu pandemic

In April 2009, the public was notified about the outbreak of a new influenza virus in Mexico. During March and April of that year, almost 2000 cases had been registered, with initial reports indicating severe illness among young and healthy people as well as high mortality rates [1, 2]. For instance, one report of patients that had been hospitalised with the virus observed that 6,5 % had become critically ill and 41 % of these died [1]. The influenza, which was popularly called “swine flu”, was caused by an A (H1N1) virus that had not been known to cause infection in humans before. The virus was later officially termed A(H1N1)pdm09 [3].

The disease proved to be as contagious as seasonal flu and quickly spread through the Americas to Europe and Asia. In June 2009 the World Health Organization (WHO) declared a pandemic based on the criterion that the transmission was intercontinental [1, 4]. However, the severity of the disease was not part of the WHO’s definition of a pandemic [1, 5]. In fact, at the time of declaring the pandemic, the WHO considered the severity of swine flu to be comparable to seasonal flu, but with the important exception that young healthy individuals seemed to be more severely affected [1, 5–7]. Nevertheless, the WHO was concerned that the virus could mutate and become more deadly, and was also aware that morbidity rates might vary across social groups and regions, as seen in previous pandemics.

### The swine flu vaccine

The unpredictable nature of the pandemic encouraged the WHO as well as regional and national health authorities to act quickly. National and supra-national pandemic preparedness plans and vaccine strategies were activated shortly after the outbreak in Mexico, and the WHO initiated a rapid process of vaccine development once it had been established that the seasonal influenza vaccine did not offer protection against the pandemic virus. This vaccine was ready for use in September 2009, after a development and production time of four and a half months [1]. By comparison, the seasonal flu vaccine is usually developed and produced on a large scale in six months. The process used was otherwise similar. The testing and authorizing process by the European Medicines Agency (EMA) and the US Food and Drug Administration (FDA) was also accelerated [1, 4, 6, 8–14].

The speed of the vaccine development process gave little time to assess the effectiveness and safety of the vaccine, and assumptions about vaccine effectiveness and safety were largely based on efficacy and safety data from the seasonal flu vaccines and from testing of mock-up vaccines using another strain of flu virus [1, 8, 15]. Efficacy and safety data from seasonal flu vaccines was generally regarded to be transferable to the H1N1 vaccine. But while the different seasonal flu vaccines are used on similar populations each year, including health care workers and the elderly, the H1N1 vaccine was used on a wider selection of the population, including small children, for which there was little existing testing or pharmacovigilance data. Representative data regarding the effectiveness and safety of the new vaccine in its target population groups was first collected through monitoring as mass vaccination was being implemented. It then became evident that the vaccine was more effective than first assumed and that at least short-term adverse effects were similar to those of the seasonal flu vaccines. In 2012, however, rare but serious long-term effects were identified in several European countries, including an association with narcolepsy in children and adolescents vaccinated with some of the pandemic vaccines that used a new type of oil-based adjuvant and a mercury-containing preservative [15]. The exact relationship between the vaccine and this chronic disorder has still not been established.

### National and international responses to the swine flu pandemic

In July 2009, the WHO recommended that all countries should begin by vaccinating their health workers. Countries were then advised to prioritise high-risk groups, including pregnant women, people with specific chronic conditions, healthy young adults, healthy children, and finally, older adults [16]. However, the WHO pointed out that the vaccines had not yet been extensively evaluated for their safety in certain population groups, and underlined the importance of post-implementation surveillance and rapid sharing of safety and effectiveness studies. The WHO also made it clear that the recommendations would need to be changed if and when new evidence became available.

National health authorities responded differently to the pandemic [1, 4, 5, 9, 17]. Most countries followed the WHO’s recommendations regarding priority groups, although there was some variation, for instance with regard to whether vaccination was recommended for small children [4]. Some wealthy countries offered vaccines to the whole population while other countries only offered vaccines to those defined as priority groups. This was primarily because some countries did not have enough vaccines for universal access, but was also because some authorities deemed the severity of the disease as low or because they were concerned about the safety of the vaccine [6, 17]. As it gradually became likely that the disease would not be as severe as first suspected, some countries also changed their vaccination strategies [1, 6]. National health authorities also used different strategies to distribute the vaccine, and bought different types of vaccines; with or without adjuvants and with different types of adjuvants and preservatives. Many European countries, as well as Canada, used the oil-based adjuvated vaccines, while for instance the USA used vaccines with traditional, aluminium-based adjuvants [15]. Several reports have pointed out that this cross-country variation in vaccination strategies was unfortunate as it was difficult for national health authorities to explain to the public why they apparently drew different conclusions from risk assessments regarding the pandemic and the vaccine than other comparable countries [1, 6, 9, 17].

### Public responses to the swine flu pandemic and to the vaccine strategies

Regardless of the strategies they chose, most governments were dissatisfied with their vaccination rates, and in 2010, only four out of 30 EU countries reported that they had met their vaccination targets [6]. Since the pandemic, a number of survey-based studies have aimed to identify factors that influenced people’s uptake of the pandemic vaccine [18–34]. These surveys, as well as two reviews of the surveys [23, 35] suggest that people balanced what they knew about their risk of getting the disease with what they knew about the safety of the vaccine, many deciding that swine flu was not severe enough to accept a vaccine of unknown safety.

In addition to these quantitative surveys, a number of qualitative studies have been carried out to understand more about people’s knowledge, attitudes and behaviour regarding the H1N1 pandemic and the vaccine. A deeper understanding of people’s reactions to pandemics and to public health recommendations can provide a good basis for future policy decisions regarding public health threats where there is much uncertainty. However, a systematic review of the qualitative studies has so far not been conducted.

## Objectives

We set out to synthesise the results of existing qualitative studies with the primary aim of exploring attitudes to the pandemic vaccine among the public in different parts of the world and belonging to different target groups. Our secondary aim was to describe and discuss the implications primary study authors had drawn from their own results.

## Methods

### Study inclusion criteria

#### Phenomena of interest

We included studies where the primary focus was the experiences and attitudes of vaccine recipients or potential recipients regarding the H1N1 influenza vaccine delivered in connection with the 2009 pandemic.

#### Types of studies

We included studies that used qualitative study designs including ethnographic research, case studies, and process evaluations. We included these studies if they had used qualitative methods for data collection, including focus group interviews, individual interviews, observation and document analysis; and qualitative methods for data analysis, including thematic analysis or any other appropriate qualitative analysis method that enables analysis of text and observations and narrative presentation of findings.

#### Study exclusion criteria

We excluded the following types of studies:

- Studies where qualitative research methods had not been used or where the qualitative data in a mixed methods study were not presented separately
- Studies where the focus was on attitudes to vaccines in general (i.e., not specifically the swine flu vaccine)
- Studies where data had been collected before 2009
- Studies where the study participants were not talking about their own views (e.g., healthcare workers or planners talking about the public’s attitudes)
- Studies where the full text was in a language we did not master

### Data collection and analysis

#### Search methods for identification of studies

We searched the following databases for studies published from April 2009 onwards:

- MEDLINE In-Process & Other Non-Indexed Citations, MEDLINE Daily, MEDLINE and Ovid OLDMEDLINE 1946 to Present, OvidSP (searched 9th December 2013)
- CINAHL 1980 to present, EbscoHost (searched 16th December 2013)
- EMBASE 1980 to 2013 Week 50, OvidSP (searched 16th December 2013)
- Psycinfo 1987 to Month 12 Week 51 2013 (searched 18th December 2013)
- Science Citation Index 1975 to present, Social Sciences Citation Index 1975 to present, ISI Web of Knowledge (Searched 18th December 2013)

We limited searches to English, Scandinavian languages and Spanish for feasibility reasons. See Additional file 1 for search strategy.

#### Study selection procedure

One review author (BC) assessed the titles and abstracts of the identified records and removed all records that were clearly not relevant. Both review authors then independently assessed the eligibility of the remaining records. The full text of all the papers identified as potentially relevant by one or both review authors were retrieved. These papers were then assessed independently by both review authors. Disagreement between review authors was resolved through discussion.

#### Analysis

We extracted data from each paper that was broadly relevant to the objectives of the review, i.e., all data related to people’s experiences and views regarding the disease, preventive measures (including the vaccine), treatments, and information about swine flu. We also extracted background information about the first author’s name, year of publication, the country where the study took place, the study participants, when the study took place and the aim of the study (See Table 1).

**Table 1:** Profile of the included studies

First author, year	Where	Who	Data source	When	Authors’ aim
Björkman, 2013 [47]	Sweden	General public Not vaccinated	Individual interviews 28 participants	Winter 2010–11	To explore motives, beliefs and reactions of individuals with varying backgrounds who did not get vaccinated.
Boerner, 2013 [53]	Canada	General public.	Focus groups and key informant interviews 130 participants	November-February 2010-11	To identify and analyse the factors related to vaccine uptake and refusal
Boyd, 2013 [43]	USA (Georgia)	Priority group (Low income women) Health care providers	6 focus groups and 10 key informant interviews 66 participants	June–August 2010	To explore knowledge, attitudes and behaviours of low-income women; improve communication in emergency response. To identify factors that affect this high priority group’s ability to successfully comply with vaccination recommendations.
Caress, 2010 [50]	UK	Priority group (Respiratory conditions)	3 focus groups and 13 individual interviews 43 participants	November 2009–January 2010	To explore and compare info needs, worries and concerns, and health-related behaviours regarding H1N1 in people with respiratory conditions and their family members
Cassady, 2012 [46]	USA (California)	Priority group (Latino hard-to-reach populations)	10 focus groups 90 participants	Summer 2010	To gather a better understanding of the dynamics that limit flu and H1N1 vaccination among hard-to-reach Latinos in California.
d’Alessandro, 2012 [45]	France	Priority group (Cyctic fibrosis patients)	Individual interviews 42 participants	June 2010	To analyse the reasons for refusal of H1N1 vaccination. Perceptions of vaccine, disease, related risks in patients that declined and accepted the vaccination
Driedger, 2013 [58]	Canada	Priority group (Aboriginal peoples)	23 focus groups and 20 individual interviews 213 participants	August 2009–June 2012	Focus on how First Nations and Metis people in Manitoba, Canada, responded to the public health management of pandemic H1N1
Henrich, 2012 [52]	Canada	General public	Online comments to news articles 1,796 commentators	March 2009–May 2010	Despite efforts to promote vaccination, the public’s intent to vaccinate remained low. In order to better understand the public’s resistance to getting vaccinated this study addressed factors influencing the public’s decisions
Hidiroglu, 2010 [49]	Turkey	Priority group (Health care providers)	Focus groups 33 participants	November 2009	To explore the factors that lead to resistance to vaccination among a group of primary healthcare workers in a district in Istanbul.
Hilton, 2010 [54]	UK	General public Priority groups (Pregnant women)	14 focus groups 73 participants	October 2009–January 2010	To gain new insights into public understandings of the role of key players in the pandemic and to explore how people deciphered the threat and perceived whether they could control the risks.
Lynch, 2012 [51]	USA	Priority group (Pregnant women)	18 focus groups 144 participants	September 2009	Presents findings from pregnant and recently pregnant women regarding their perceptions about the 2009 H1N1 and seasonal flu vaccines. The paper further identifies needed info to improve communication strategies to encourage the H1N1 and seasonal flu vaccine and potentially future pandemic vaccines.
Oria, 2011 [55]	Kenya	Priority group (Health care providers)	16 focus groups 113 participants	January 2010	To characterize health care providers HCWs’ knowledge, attitudes and practices regarding pH1N1 vaccination,
Prieto Rodríguez, 2009 [57]	Spain	General public Priority groups Health care providers	10 focus groups 51 participants	January–February 2010	To identify opinions of the general population, risk groups and health care providers of the 2009-repercussions.
Sim, 2011 [44]	UK	Priority group (Pregnant women)	Individual interviews 10 participants	November 2009	To assess how pregnant Polish migrants to Scotland weighed up the risks and benefits of the V for pandemic H1N1 flu in comparison with their Scottish counterparts.
Siu, 2012 [56]	China (Hong Kong)	Priority group (Chronic renal disease patients) Not vaccinated	Individual interviews 40 participants	December 2009–March 2010	To demonstrate the perceptions of patients with chronic renal disease in Hong Kong towards the new vaccine for H1N1, as well as the main disincentives.
Teasdale, 2011 [48]	UK	General public	11 focus groups 48 participants	September–November 2009	To explore people’s beliefs, perceptions, reasoning, and emotional and contextual factors that may influence responses to government advice for managing flu pandemics

When analysing the data we used a broadly comparative case study approach informed by tools and techniques outlined in the narrative synthesis framework, where each included study was considered a case [36]. In line with this approach, both review authors independently read and reread the selected studies, and identified key themes, much as we do when analysing interview transcripts or other types of documents in primary qualitative research. The review authors searched for themes in all sections of the reports, including the background, results and discussion sections until all the studies were accounted for and no new themes were identified. The review authors then discussed and agreed upon the definitions and boundaries of each of the emerging themes and then grouped them into broader themes. These themes, in combination with a list of included studies were used as the basis for a matrix derived from an approach described by Miles and Huberman [37]. Both review authors then extracted and condensed the information from each study pertaining to the different themes and inserted this information into the matrix. The matrix also summarised key information about each study (first author, date, country, study characteristics), and facilitated detailed comparison of each theme across the studies, constituting the basis for our synthesis of findings from the studies. By re-arranging the order of the studies according to the different background characteristics, e.g., study country, type of participants, time of study, the review authors were also able to check whether there were any patterns in the findings related to these traits.

#### Assessment of methodological limitations in the included studies

Both review authors independently applied a set of quality criteria to each included study. Any disagreements between the two review authors were then resolved through discussion. The review authors appraised the studies using the main elements of the Critical Appraisal Skills Programme (CASP) quality assessment tool for qualitative studies [38], as in other syntheses of qualitative evidence [39–41]. We did not exclude studies because of low study quality.

The main assessment criteria extracted from CASP were:

1. Is the study context clearly described?
2. Is the sampling method clearly described and appropriate for the research question?
3. Is the method of data collection clearly described and appropriate for the research question?
4. Is the method of data analysis clearly described and appropriate for the research question
5. Is there evidence of researcher reflexivity?
6. Are the claims made supported by sufficient evidence?

(For an overview of the quality assessment, see Additional file 2.)

## Results

### Description of the included studies

#### Results of the search

**Fig. 1:**
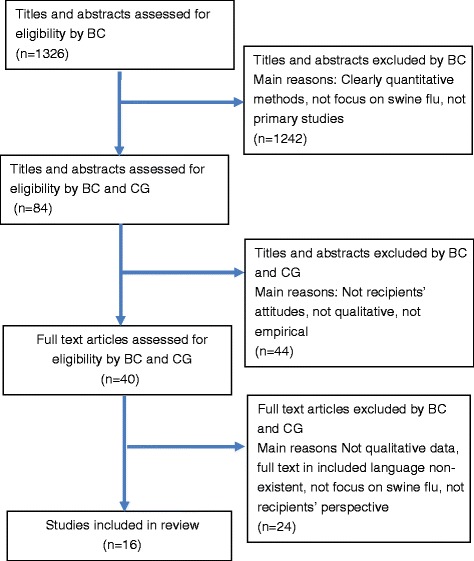
Flow chart. Overview over search and selection process leading to final sample of 16 included studies

We initially identified and assessed 1326 titles and abstracts and considered 40 of these studies in full text. We assessed sixteen of the studies as fulfilling our inclusion criteria and included these in the review (See Fig. 1-Flow chart).

#### Included studies-methods, setting, participants and timeframe

Among the 16 included studies, seven gathered data through focus group interviews, four used individual interviews and four combined focus groups and individual interviews, while one study analysed online comments to news articles. Four studies were from the UK, three from Canada, three from the USA and one from China (Hong Kong), France, Kenya, Spain, Sweden and Turkey respectively. Ten of the studies studied high risk or priority groups, including healthcare providers, pregnant women and people with chronic respiratory conditions; four studies studied the general public; and two studies studied both priority groups and the general public. Two studies focused on individuals that had chosen not to get vaccinated, while the remaining studies included people who had made different decisions. Most of the data collection took place during the swine flu pandemic from 2009 to 2010, and before the WHO declared that the pandemic was over on August 10th 2010 [42].

(For an overview of the included studies see Table 1.)

#### Primary study authors’ aims and study premises

As specified in our inclusion criteria, all sixteen studies explicitly aimed to explore the public’s views on the pandemic influenza vaccination. For most primary study authors, achieving a better understanding of people’s perceptions was seen as a means to the goal of increasing public compliance with government vaccination plans in the future, as illustrated by the following quote:

*Researchers sought to identify the factors that affect this high-priority population’s ability to successfully comply with vaccination recommendations* [43].*:852*

Primary study authors typically described swine flu as an important threat to public health, stated that vaccination is the most effective method for preventing the disease and related complications, and referred to national influenza vaccination coverage as insufficient.

### Main themes identified in the studies

We initially identified six themes. In this paper, we will focus on three main themes that were discussed in all the included studies and that were the most relevant to our focus on people’s attitudes to vaccination. These three themes were also most relevant to the main aim of the primary studies; to explore how vaccination goals could be achieved in future epidemics. These three themes are people’s perceptions of the disease’s severity and spread, their perceptions of the vaccine, and their perceptions of health authorities’ information and vaccination strategies.

The additional three themes that will not be discussed here included people’s perceptions of the information they received from the media; their perceptions of the information they received from health professionals; and organizational or practical barriers and facilitators to vaccination.

#### People’s perceptions of disease severity and spread

Some participants questioned the extent to which the H1N1 virus really represented a pandemic or a “true emergency”, referring to the fact that their own personal experiences did not confirm this impression with regard to either severity or spread [44–46]. Most participants regarded the disease as relatively mild and as similar to other types of influenza [47–52], felt that the danger or risk was exaggerated, and did not take the government’s urgent messages seriously [44–50, 52–55]. However, others saw the disease as more severe [54, 55] or potentially serious and sometimes deadly [43, 52, 53], and referred to the relatively high risk to young people [43, 52].

In some studies, people perceived their personal risk of getting the disease as low [47, 53], while participants in other studies saw this risk as high [49] or this perception varied between participants [45, 51]. People’s perceptions about personal risk appeared to be based on assumptions about the disease itself and the extent to which it was contagious [47]; their own pre-existing health conditions [44, 51, 53, 54]; their age [53]; or the extent to which they knew of other people in their environment who had the disease [53, 55]. Some of the studies specifically described or discussed how these different perceptions regarding disease spread and severity or personal risk influenced people’s decisions to take the H1N1 vaccine [43–47, 51, 53, 56].

#### People’s perceptions of the vaccine

Study participants were particularly concerned about the vaccine’s potential side-effects. Some of this concern reflected a scepticism to vaccines in general [47, 56], but was in large part tied to concerns around the novelty of this particular vaccine and the speed in which it had been developed and approved [44, 45, 47–51, 54, 56]. Participants were concerned that the vaccine had not been sufficiently tested [43, 45, 47, 48, 52–54, 57]. In some studies participants noted that that the vaccine was in fact being tested on the public [49, 55], describing themselves as “lab rats” [46] or “guinea pigs” [44, 45, 52, 58].

Participants with serious health conditions were concerned that the vaccine would impact on these conditions or interact with other medications [50, 56]. Pregnant women pointed out that the use of the vaccine during pregnancy contradicted usual advice to avoid medication during pregnancy [44, 54]. For this group, the lack of data about potential side effects to the foetus was of particular concern [44, 51, 54]. In other studies, participants referred to a lack of information about the vaccine components [55], or they were concerned about specific components such as the adjuvants used in some of the pandemic vaccines [45, 52]. While there was most concern about the vaccine’s potential side-effects, participants in some studies also raised concerns about the vaccine’s effectiveness [43, 45, 47–49, 55].

Participants who had decided not to get vaccinated generally believed they had made the right decision [47]. One study underlined that there were no signs that those who chose not to get vaccinated had failed to understand the official messages [44].

#### People’s perceptions of health authorities’ information and vaccination strategies

In general, study participants reported that they had received inconsistent or even contradictory information from different information sources or at different points in time. This led to confusion and made some people doubt the information [43–45, 47, 49, 51, 53, 56, 57]. Pregnant women also experienced a contradiction between advice from health authorities to be cautious with medicines and their advice to accept the poorly tested pandemic vaccine [44].

Participants with unanswered questions would have liked one trusted official source of information and would have preferred to receive more information directly from the health authorities [43, 51, 57]. In some studies where priority groups were interviewed, participants wanted targeted information and a specialised information channel [50, 57].

Trust in health authorities and support for their vaccination strategies appeared to vary across study countries. UK participants typically saw the health authorities as a key information source that they generally trusted. Some of these participants stated that the pandemic had not been overhyped by the government given its potential impact and that the authorities had handled the situation well. Participants here found it reassuring that the government ordered enough vaccines and also that they decided not to use them after all [44, 50, 54]. Some participants even recognised the government’s dilemma, stating that they were “damned if they do and damned if they don’t” offer the vaccine to the population [54]. In Kenya, participants also felt that national health authorities offered clear and trustworthy information, although they did not trust international health organisations such as the WHO [55].

In Canada, participants conveyed less trust in health authorities and in two of the three studies, participants described health authorities as incompetent, accusing them of taking too little and wrong action [52, 58]. Some also thought that the authorities’ vaccination strategy was politically motivated and that they focused on minimizing absenteeism or had given in to pressure from the pharmaceutical industry instead of focusing on benefits to the public.

In the study from Hong Kong, people voiced a general distrust in health authorities which appeared to have impacted on people’s perception of vaccine safety. One participant believed that the government had continued to promote vaccination once the vaccines had been bought to avoid losing face and being blamed by the public for wasting taxpayers’ money [56].

In some studies from the USA, Kenya, Turkey, Spain, Canada and Sweden, a few participants were reported to believe in rumours or “conspiracy theories”. This included theories that the use of the vaccine was driven by the economic interests of the pharmaceutical industry [47, 52, 57], that the virus had been produced intentionally to bring economic benefits to this industry [49]; or that it was being used to distract people from the economic recession or to defame Mexican people [46]. A few had heard that the vaccine itself could cause the influenza [43] while others were suspicious about the motives behind their status as a priority group [58].

### Primary study authors’ conclusions and advice to authorities

As described earlier, the motivation of all of the primary study authors was to explore people’s perceptions of the swine flu vaccine. For most of the primary study authors this was presented as a means to the goal of increasing vaccine compliance, and all of the authors discussed the implications that health authorities could draw from the data to support this goal.

#### Increased compliance through better information and communication strategies

Based on their findings that people had doubts about the prevalence and seriousness of the disease as well as concerns about the potential side effects of the vaccine, several of the primary study authors concluded that the public was confused [50, 51, 54, 57] and misinformed [46, 51, 53]. Thus the main implication that most primary study authors drew from their findings was the need for better information or communication strategies [43, 45, 46, 48–51, 53–57], the underlying assumption being that a lack of appropriate information was the main barrier to vaccine uptake:

*- the confusion over the potential severity of H1N1 infection in pregnant women and its relationship to seasonal flu needs to be considered. (…) Patients’ concern, confusion and lack of knowledge regarding H1N1 and their willingness to change initial views highlight a critical role for education* [51].: p1662–3).

Some primary study authors encouraged health authorities to provide information about the seriousness of the disease and the need for the vaccine [49], about the reason why specific groups were targeted [58] and about the vaccine’s safety and effectiveness [43–45, 48, 49, 55, 56]. Typically, primary study authors underlined the need to address people’s varying concerns regarding the safety of the vaccine, implying that the provision of such information would lead to increased vaccine compliance:

*Constructing advice messages that address people’s beliefs and concerns is also likely to be important to persuade and empower people to adopt recommended behaviours in a pandemic* [48]:p 414)

Primary study authors also offered recommendations regarding how information about the disease and the vaccine should be communicated, for instance emphasising the importance of presenting the information clearly [45] and in plain language [50], or encouraging the authorities to offer information that was detailed [56] and evidence-based [49]. One study emphasised the need for the information to be consistent, although recognising that it may be particularly difficult to offer consistent information during a pandemic “where the situation is constantly changing and being reviewed” ( [50]:p41).

Several primary study authors also stressed that information should be delivered by what was perceived by the public to be “trustworthy”, “trusted” or “credible” sources [45, 46, 50, 51, 55, 58]. One study that recognized the importance of trust in health authorities, did not discuss how to achieve this, simply advising the authorities to improve “the public’s perception of government trustworthiness and competence” ( [52]: p11). Another study voiced the need to help people decide which information to trust [50].

While most authors focused on information provision as a key solution to low uptake, a few authors also underlined the need to involve people in the decision making process [47, 49]). The concept of “informed choice” was referred to by one team of authors [53], while other authors advised health authorities to involve the public, for instance through community-based dialogues about the vaccination campaign [49, 58], and to “address the recipients’ perspective” [48, 54]. Authors appeared to view public participation, informed choice, and the use of evidence-based information as a means to increase vaccine uptake rather than a means to encourage patient choice:

*By eliciting and addressing the perspectives of recipients of pandemic flu advice (…) future government recommendations could enable people to make choices that reduce the impact of a pandemic on themselves and society* [48]:p417).

As described above, many primary study authors regarded participants as uninformed and focused on the need to provide the public with more information and consistent messages. Very few problematized this issue [44, 58], for instance by discussing what this additional information would contain and how this information could be made clear and consistent given the uncertainty surrounding both the disease and the vaccine. One study, however, differed significantly from the others. In their study of pregnant women in the UK, Sim et al. argue that their participants had not misunderstood the information from health authorities, but had in fact internalised the rationale used in previous public health messages, particularly in connection with the MMR vaccine. For MMR, the public had been reassured of the safety of the vaccine by referring to the existence of robust clinical trials with long-term follow-up. “*It was exactly this type of scientific reasoning that women drew on in highlighting what they perceived as the absence of such information on the H1N1 vaccine, and their consequent uncertainty about whether or not to accept it*” [44]:510. These study authors argued that health authorities in future situations should be open and transparent about uncertainty:

*Explicitly addressing uncertainty and acknowledging areas where there has been no opportunity to accumulate evidence may serve to enhance rather than diminish the credibility of face-to-face or published material which enables informed choice as the influenza vaccine is offered routinely to pregnant women in the UK provides a potential opportunity to address these wider and more challenging issues in the development of health information* [44].: p510)

### Quality appraisal of the included studies

All of the studies gave some description of the strategies they had used to select participants and to collect and analyse data. Although these descriptions tended to be brief, we assessed these strategies as appropriate to the research question.

However, two other elements of the quality assessment gave us reason for concern. First, there was a lack of contextual information in almost all of the studies, particularly regarding the type of information that the authorities, the public in general, and study participants in particular had access to during the pandemic. Exceptions to this were seen in three studies where primary study authors described the type and quality of information available to the public through the media [44, 50, 54, 58]. Secondly, there was no evidence of researcher reflexivity in any of the studies. Reflexivity is perceived as an integral process in qualitative research and requires that researchers reflect upon their own background and position, and how it will affect “what they choose to investigate, the angle of investigation, the methods judged most adequate for this purpose, the findings considered most appropriate, and the framing and communication of conclusions” [58]:p483–4. This can entail, for instance, reflecting on how the researcher’s status and role may have influenced what the informants disclose and how it is presented to the researcher [59]. We found no explicit discussion of the primary study authors’ background or position, including how their studies might have been influenced by these factors, nor were there any information about their knowledge or opinions of issues such as the pandemic, the vaccine, or the role of the individual decision maker when faced with potential public health crises.

These two weaknesses led us to question a third element in the quality assessment, that is, the extent to which the claims made by the study authors were sufficiently supported by the evidence. We will return to these issues in our discussion below.

## Discussion

The studies that we identified in our review describe how people had varying perceptions about the disease and the vaccine, but were often uncertain about the severity of the disease, the spread of the disease, and any risks tied to the vaccine. People who chose not to be vaccinated usually perceived their individual risk of getting the disease as low, believed that they would not get very sick from it, or were concerned that the vaccine could lead to side effects. These findings are very similar to findings from a number of quantitative surveys, reviews and official evaluation reports [1, 6, 17, 18, 20–23, 25, 30, 31, 33, 34, 59].

### Did primary study authors draw the right conclusions?

Most of the primary study authors concluded that participants were uninformed, and that more information about disease severity and vaccine safety and effectiveness would lead to an increase in vaccine uptake. We find this conclusion problematic. Most primary study authors offered very little contextual information about what evidence regarding the pandemic and the vaccine that was available to authorities and to members of the public, or about the certainty of this evidence. Solely based on the included papers, we were therefore unable to properly assess primary study authors’ claims that the uncertainties participants described often were a result of poor information. We thus tried, through other sources (including the research literature, official reports and websites referred to in our background chapter), to get a better picture of the knowledge authorities had at the time, as well as the information that was available to the public during the pandemic. This information suggests that while some participants probably did miss or misunderstand information, most of the uncertainties described by study participants reflect those held by health authorities and scientific environments at the time, both with regard to disease severity, disease spread, and potential vaccine side-effects. In fact, one might even suggest that participants’ concerns were a rational response to the information they received from the authorities rather than the result of a lack of information. With the exception of Sim et al. [44], and to a lesser degree Caress et al. [50], none of the primary study authors discussed the fact that the authorities were also uncertain about disease spread and severity or vaccine benefits and harms, particularly for certain parts of the population.

Health authorities have different decision making roles than individuals. While health authorities are responsible for the well-being of the public as a whole, individuals are concerned with their own health and the health of their families. But despite these differences, during the swine flu, national authorities found themselves in a similar position to that of the study participants as both groups were faced with the dilemma of choosing action or inaction without knowing the full implications of either choice. This comparison was not made by most primary study authors. Instead, the authors focus on the authorities’ decisions to implement vaccination programmes, making little or no reference to the processes and assessments of uncertainty leading up to these decisions. Primary study authors appear to assume that the vaccine was effective and safe and that the pandemic represented a serious threat, something that neither the public, the primary study authors, nor the authorities could know with confidence at that time.

We regard this lack of information about and reflection on the context of the studies as a weakness in the studies and connect it to a lack of researcher reflexivity demonstrated by the primary study authors. The authors’ contact information indicates that the majority of them held positions at medical faculties and that some of them were Ministry of Health employees. This suggests that they have approached their own study with a public health perspective, focusing on the achievement of herd immunity through patient compliance. The primary study authors’ aim of increasing vaccine compliance appears to have influenced their recommendations that the public should be given more information and more consistent information, while ignoring any uncertainties this information might contain. Although a few authors do refer to concepts such as shared decision-making, informed choice and public involvement, they do not discuss the potential tension between these approaches and their goal of increasing vaccine compliance, illustrated by one primary study author’s contradictory statement that the public should be both “empowered and persuaded” [48]. The uncertainty which is a typical feature of pandemic vaccine development [15, 60] can increase this tension between public and individual interests. When faced with a potential public health crisis and under pressure to act quickly, authorities are probably far less inclined to highlight uncertainties or to practice a transparent decision-making process for fear that this will slow down or prevent vaccine uptake, and the tension between public health goals and the goals of the individual and of shared decision making can therefore be intensified. At the same time, members of the public are also likely to be more skeptical to the safety of vaccines that have been developed, approved and distributed by authorities in haste.

### What other conclusions could the primary study authors have drawn?

An alternative conclusion that primary study authors might have drawn, and that we think would have been more in line with their own findings, would have been to recommend more transparency regarding uncertainty as a strategy for increasing public trust in health authorities and thereby compliance with public health strategies.

One of the included studies do advocate transparency from health authorities in situations of uncertainty [44]. This study is in line with several research reports that argue for transparency in public health crises of “radical uncertainty”, such as pandemics [60, 61]. In the aftermath of this pandemic, health authorities were criticised for a lack of transparency with regard to decision making processes and the evidence base for these decisions [6, 60, 62–64]. A report evaluating the WHO’s performance during the pandemic also advises the WHO and other health authorities to practice transparency in future pandemic situations [1].

There are a number of reasons why transparency in the face of uncertainty might be a sensible strategy [60, 61]. First of all, openness regarding core public health objectives, such as herd immunity, may lead to an acknowledgement and acceptance of these aims among the public. Also, in situations where people suspect uncertainties regarding the spread or severity of the disease or the safety and effectiveness of vaccines, authorities’ openness about levels of uncertainty may help address conspiracy theories. This would be difficult in situations where uncertainty is downplayed. Another reason is that initial assumptions may turn out to be wrong. In this case, the pandemic was less dangerous than first feared, and the vaccine is now associated with rare but serious side-effects. If health authorities downplay uncertainties, it becomes hard to explain any changes in government strategies when new knowledge emerge and this can potentially undermine the public’s willingness to trust information from health authorities in the future. According to an EU report evaluating authorities’ management of the swine flu, transparency “about what is not known is just as important to the promotion of public trust as transparency about what is known. Trust requires honest, open and two-way communication” ( [61]: p615).

The UK studies in this review suggest that the public can handle the knowledge that health authorities make decisions in the face of uncertainties and that these decisions may be wrong. Here, study participants reported more positive attitudes to the government’s handling of the pandemic than most others, and found it reassuring that the government ordered enough vaccines but decided not to use them after all [44, 50, 54], suggesting that the acknowledgement of uncertainty and changes in strategy are not necessarily perceived negatively by members of the public.

While changes in strategy appeared to be acceptable to at least some participants, participants in several studies called for consistent information. The importance of consistency was also emphasised in recommendations made by several primary study authors and is supported by a European Commission report on the EU-wide pandemic vaccine strategies [65]. Here, the European Commission concludes that health authorities should strive to report consistent health messages across countries “to ensure citizens do not receive mixed or wrong messages depending on the area they are in” ( [65]:p51). But is it possible to be transparent about uncertainties while also offering information that is consistent? An independent review of the UK pandemic response concludes that health authorities managed to deliver a comprehensive information campaign which combined the need for clarity and a “single authoritative voice” with recognition of and continuous updating on the uncertainty of the disease ( [59]:p134). In this approach, consistency is achieved by ensuring that all of the population receives the same message from the same source, while uncertainty and the fact that information can change over time is acknowledged. In other words, information needs to be consistent at any given time, but this does not imply that it cannot change over time.

The success of a transparent approach in these types of situations is likely to depend on the relationship between the authorities and the public more generally. In addition, studies of shared decision making in primary health care suggest that members of the public may not always want to make health care decisions on their own or to share the responsibility for a decision if it turns out to have negative implications [66–68].

But while transparency is not unproblematic, keeping information from the public may not be an option in most societies. An increasing number of people have access to a broad spectre of information across borders. The public can be expected to find out about inconsistent policies across countries or about scientific uncertainty, or worse, may be misguided by speculations and rumours. Attempts from authorities to hide uncertainties may thereby boost distrust and conspiracy theories. It is therefore hard to think of any better strategy than for governments to be transparent about clinical and political processes as well as the reasons behind national strategies.

#### Strengths and limitations of the review

Obviously, our own backgrounds and positions have influenced our interpretations of the studies, and this may in different ways have led us to focus more than the primary study authors on the uncertainties faced by the authorities surrounding the pandemic and the vaccine. While we are both social scientists without expert knowledge about the pandemic or the vaccine, we had the advantage of hindsight regarding the actual prevalence of the pandemic and the longer-term effects of the vaccine. This may have made it easier for us to question the knowledge that was available to health authorities at the time.

Our synthesis of existing studies gave us access to data from a large number of participants and several different countries. The review showed that there were both commonalities and variations in people’s attitudes to the swine flu vaccine and allowed us to look for patterns in this variation by rearranging the matrix according to characteristics of the study or the participants. The only consistent pattern we discovered, however, was a variation across countries regarding people’s views of government information and strategies, as described above. While these differences are interesting the range of study countries in the review and the number of studies from each country is too small and the reasons behind variations in people’s trust in their national government too complex to draw robust conclusions about the registered differences. Thus, it appears that a main limitation of this review is the small number and skewed range of study countries. Most of the studies were from Western countries and from countries with democratic governments, there were no studies from Latin America (where the pandemic originated), and few were from low income countries.

It is possible that people’s views about the information they receive from health authorities are influenced by the form of government in their country, while people’s understanding of disease and of disease prevention and treatment is likely to be influenced by their level of education. For instance, people in some countries may be more likely to trust authorities and comply with vaccination advice without doubting or questioning the rationale behind it. Our suggestion that transparency about uncertainty in pandemics is a necessary and inevitable strategy may therefore not be transferable to every national context.

## Conclusion

While the authors of the primary studies included in this review suggest that national health authorities could increase vaccine uptake through increased and more consistent information, we suggest instead that health authorities should be more transparent in their information and decision-making processes in future pandemic situations.

In the future, researchers should offer more information about study context and pay more attention to issues of reflexivity to ensure the relevance, trustworthiness and usefulness of their advice for policy makers and others.

## Additional files

Additional file 1: Search strategies. Overview over search history; words and combinations in the different databases we used. (PDF 400 kb) Additional file 2: CASP. Overview of quality assessment (CASP). (PDF 191 kb)
