# Supplementary material for: The swine flu vaccine, public attitudes, and researcher interpretations: a systematic review of qualitative research
Source: BMC Health Serv Res. 2016 Jun 24;16:203. doi: 10.1186/s12913-016-1466-7 (PMC4919843; doi:10.1186/s12913-016-1466-7)
Supplement: Additional file 1: — Search strategies. Overview over search history; words and combinations in the different databases we used. (PDF 400 kb) [file 12913_2016_1466_MOESM1_ESM.pdf]

Additional file 1: Search strategies

Ovid Medline (9th Dec 2013)

1	Immunization/
2	Immunization Programs/
3	Immunization, Schedule/
4	Vaccination/
5	Mass Vaccination/
6	Vaccines/
7	Influenza vaccines/
8	(vaccine* or immuniz* or immunis*).ti,ab.
9	or/1-8
10	Influenza A Virus, H1N1 Subtype/
11	(swine flu or H1N1 or swine influenza).ti,ab.
12	Or/10-11
13	9 and 12
14	limit 13 to "qualitative (maximizes sensitivity)"
15	Qualitative Research/ or Interviews as Topic/ or (qualitative or group discussion? or focus group? or themes).ti,ab.
16	13 and 15
17	14 or 16
18	limit 17 to (danish or english or spanish or norwegian or swedish)
19	(2009 or 2010 or 2011 or 2012 or 2013).yr.
20	18 and 19

Search strategy used for Embase (16th Dec 2013)

1	immunization/
2	mass immunization/
3	vaccination/
4	revaccination/
5	vaccine/
6	or/1-5
7	Influenza virus A H1N1/
8	(swine flu or H1N1 or swine influenza).ti,ab.
9	or/7-8
10	6 and 9
11	Limit 10 to "qualitative (maximizes sensitivity)"
12	qualitative research/ or interview/ or (group discussion? or focus group? or themes).ti,ab.
13	10 and 12
14	11 or 13
15	limit 14 to (danish or english or spanish or norwegian or swedish)
16	(2009 or 2010 or 2011 or 2012 or 2013).yr.
17	15 and 16
18	limit 17 to embase

Search strategy used for Cinahl (16th Dec 2013)

1	(MH “Immunization”)
2	(MH “vaccines”)
3	TI (immun* or vaccin*) OR AB (immune* or vaccin*)
4	1 or 2 or 3
5	(MH “Influenza A Virus, H1N1 Subtype”)
6	(MH “Influenza, Pandemic (H1N1)”)
7	TI (swine flu or swine influenza or h1n1) OR AB (swine flu or swine influenza or h1n1)
8	5 or 6 or 7
9	4 and 8
10	(MH "qualitative studies")
11	TI (qualitative research or interview or group discussion* or focus group* or themes) OR AB (qualitative research or interview or group discussion* or focus group* or themes)
12	10 or 11
13	9 and 12
14	Limit to articles published from 2009 to 2013
15	Limit to articles published in academic journals

Search strategy used for Ovid Psycinfo (18th Dec 2013)

1	Immunization/
2	Vaccination/
3	(vaccine* or immuniz* or immunis*).ti,ab.
4	or/1-3
5	(swine flu or H1N1 or swine influenza).ti,ab.
6	4 and 5
7	Qualitative Research/ or Interviews as Topic/ or (qualitative or group discussion? or focus group? or themes).ti,ab.
8	6 and 7

Search strategy used for Science Citation (18th Dec 2013)

1	Immuniz*
2	Immunis*
3	Vaccin*
4	1 or 2 or 3
5	“swine flu”
6	H1N1
7	5 or 6
8	4 and 7
9	“Qualitative Research”
10	Interviews
11	“group discussion”
12	“focus group”
13	themes
14	9 or 10 or 11 or 12 or 13
15	8 and 14
16	Only 2009 - 2013
