# Supplementary material for: The swine flu vaccine, public attitudes, and researcher interpretations: a systematic review of qualitative research
Source: BMC Health Serv Res. 2016 Jun 24;16:203. doi: 10.1186/s12913-016-1466-7 (PMC4919843; doi:10.1186/s12913-016-1466-7)
Supplement: Additional file 2: — CASP. Overview of quality assessment (CASP). (PDF 191 kb) [file 12913_2016_1466_MOESM2_ESM.pdf]

Author year	Is the context clearly described?	Is there evidence of researcher reflexivity?	Is the sampling strategy clearly described and appropriate?	Is the method of data collection clearly described and appropriate for the research question?	Is the method of data analysis clearly described and appropriate for the research question?	Are the claims made supported by sufficient evidence?
Bjorkman 2013	Partly	No	Yes	Yes	Yes	Partly
Boerner 2013	Partly	No	Yes	Partly	Partly	Partly
Boyd 2013	Partly	No	Yes	Yes	Yes	Partly
Caress 2010	Yes	No	Partly	Partly	Partly	Partly
Cassady 2012	Partly	No	Partly	Yes	Yes	Partly
D'Alessandro 2012	Partly	No	Partly	Partly	Partly	No
Driedger 2013	Yes	No	Yes	Yes	Yes	Yes
Henrich 2012	Partly	No	Partly	Yes	Yes	Yes
Hidiroglu 2010	Partly	No	Partly	Partly	Partly	No
Hilton 2010	Yes	No	Yes	Yes	Yes	Yes
Lynch 2012	No	No	Partly	Yes	Partly	Partly
Oria, 2011	Partly	No	Partly	Partly	Partly	Yes
Rodriguez, 2009	Partly	No	Yes	Partly	Partly	Yes
Sim 2011	Yes	No	Partly	Yes	Yes	Yes
Siu 2012	Partly	No	Yes	Yes	Yes	Partly
Teasdale, 2011	No	No	Yes	Yes	Yes	Partly
